# Epibrassinolide activates AKT to trigger autophagy with polyamine metabolism in SW480 and DLD-1 colon cancer cell lines

**DOI:** 10.3906/biy-2005-37

**Published:** 2020-12-14

**Authors:** Kaan ADACAN, Pınar OBAKAN YERLİKAYA

**Affiliations:** 1 Department of Molecular Biology and Genetics, Science and Literature Faculty, İstanbul Kültür University, İstanbul Turkey

**Keywords:** Autophagy, epibrassinolide, polyamines, LC3, spermidine

## Abstract

Epibrassinolide (EBR), a plant-derived polyhydroxylated derivative of 5α-cholestane, structurally shows similarities to animal steroid hormones. According to the present study, EBR treatment triggered a significant stress response via activating ER stress, autophagy, and apoptosis in cancer cells. EBR could also increase Akt phosphorylation in vitro. While the activation of Akt resulted in cellular metabolic activation in normal cells to proceed with cell survival, a rapid stress response was induced in cancer cells to reduce survival. Therefore, Akt as a mediator of cellular survival and death decision pathways is a crucial target in cancer cells. In this study, we determined that EBR induces stress responses through activating Akt, which reduced the mTOR complex I (mTORC1) activation in SW480 and DLD-1 colon cancer cells. As a consequence, EBR triggered macroautophagy and led to lipidation of LC3 most efficiently in SW480 cells. The cotreatment of spermidine (Spd) with EBR increased lipidation of LC3 synergistically in both cell lines. We also found that EBR promoted polyamine catabolism in SW480 cells. The retention of polyamine biosynthesis was remarkable following EBR treatment. We suggested that EBR-mediated Akt activation might determine the downstream cellular stress responses to induce autophagy related to polyamines.

## 1. Introduction

Epibrassinolide (EBR), a member of brassinosteroid plant hormones with structural similarities to mammalian steroid hormones, promotes plant growth and has antioxidant properties. Recently, its apoptosis-promoting roles have been identified in prostate and colon cancer cells (Obakan et al., 2014; Coskun et al., 2015; Obakan-Yerlikaya et al., 2017). Although EBR was first suggested as a nuclear hormone receptor (NHR) inhibitor due to its steroid-like structure, it was also able to induce cell death in non-NHR expressing cancer cells, which conducted the research to a different path. Our studies have shown that EBR is actually an endoplasmic reticulum (ER) stress inducer which was first identified with stable isotope labeling by amino acids in cell culture (SILAC) (Obakan et al., 2015) and mass spectroscopy analyses. Our following publication suggested that EBR induced chronic ER stress to induce several signaling mechanisms (Obakan-Yerlikaya et al., 2017), especially those playing a role to induce apoptosis. ER stress is a process during which cells tend to die due to improper function of unfolded/misfolded proteins. One of the cellular signaling pathways induced by ER stress is autophagy. This critical cellular process has been found to be activated as a defense mechanism to maintain cell survival (Cybulsky, 2017; Song et al., 2018).

During autophagy, the degradation of misfolded proteins, aged organelles, and macromolecules occurs. In addition, stress and energy deprivation can induce autophagy in a programmed way with the involvement of autophagy-regulating proteins. The molecular mechanisms of autophagy defects have been related to various diseases including aging, neurodegeneration, and cancer (Akkoç and Gözüaçık, 2018; Kocaturk et al., 2019). Tumor cells can use autophagy as a survival mechanism. The degradation of faulty organelles and misfolded proteins provides resistance to anticancer therapies (Gozuacik and Kimchi, 2004). However, under extreme stress conditions, tumor cells can go through autophagy-induced cell death, also known as type 2 PCD (programmed cell death) characterized by an irrepressible increase of autophagic flux. Autophagy is induced through the Akt/mTOR signaling pathway and regulated by autophagy-related genes (ATGs) (Gozuacik and Kimchi; 2007). Previous studies showed that a variety of different brassinosteroid family members, which include 6-keto group and 22α,23α-hydroxyls,exerted anabolic activity and activated Akt to improve metabolism in muscular cells (Esposito et al., 2011). Similar to Akt, the potential EBR-mediated alterations are important in the induction of autophagy. Therefore, the clarification of potential drug candidates and critical cellular targets in cancer cells are important to evaluate autophagy-mediated successful therapeutic modalities (Cybulsky, 2017).

Polyamines are organic cations that play roles in cell proliferation, differentiation, and survival. Putrescine (Put), spermidine (Spd), and spermine (Spm) are aliphatic amines synthesized in sequence from decarboxylation of ornithine (Eisenberg et al., 2009). Polyamines consist of a flexible polycationic structure that allows them to bind electrostatically to negatively charged molecules such as nucleic acids, acidic proteins, and membranes (Palavan-Unsal et al., 2006). Significant increases of polyamine levels and their acetylated forms in cancer patients are reported and associated with rapidly growing and proliferating tumor cells. Spermidine prolongs the lifespan of several organisms such as nematodes, yeasts, and flies (Yue et al., 2017). In addition, Spd induces autophagy in these organisms as well. However, with the genetic or pharmacological extinguishing of autophagy-related genes, these organisms lose beneficial effects such as a prolonged life span (Yue et al., 2017). This indicates that the cytoprotective/antiaging effect of Spd is dependent on autophagy and chromatin-mediated regulation of gene expression (Hai et al., 2017).

## 2. Materials and methods

### 2.1. Cell culture

Human colon cancer cell lines SW480 (CCL228) and DLD-1 (CCL221) were purchased from the American Type Culture Collection (ATCC). The DLD-1 cells were grown in McCoy’s 5A medium (PAN-Biotech GmbH, Aidenbach, Germany), and the SW480 cells were grown in MEM medium (PAN-Biotech GmbH). All the media were supplemented with 10% fetal bovine serum (PAN Biotech), 1000 U penicillin/mL, and 10 mg streptomycin/ml (PAN-Biotech GmbH). The cells were cultured at 37 °C in humidified 5% CO2 incubator (HERA cell 150; Thermo Electron Corporation, Waltham, MA, USA).

### 2.2. Immunoblotting analysis

The cells were cultured in 60 mm Petri dishes in complete medium and treated with drugs. The cells were washed and collected with 4 °C 1X PBS twice and lysed in M-PER (Thermo Fisher Scientific Inc., Waltham, MA, USA) for 20 min. Then, the lysate was centrifuged for 15 min at 16,000 rcf. Supernatants were retained and stored at −80 °C for further experiments. Protein concentrations were calculated with the Bradford assay. Then, protein extracts were prepared with 5× Laemmli buffer and 45 µg proteins for every condition were loaded on 12% SDS-PAGE and transferred to PVDF membranes. The membranes were blocked with 5% nonfat milk in 0.1% TBST for 1 h and incubated with primary antibodies against anti-Akt, anti-p-Akt ser473, anti-p-PDK-1 ser 243, anti-mTOR, anti-p-mTOR ser2448, anti-PAO, anti-SSAT, anti-ODC, anti-AZ, and anti-AZI overnight at 4 °C, followed by incubation with appropriate secondary antibodies. Equal lane loading was confirmed using a monoclonal antibody against b-Actin and GAPDH. After washing with the TBS-T buffer, the membranes were incubated with ECL solution for 2 min and scanned with chemiluminescence reader (Biorad ChemiDoc XRS+).

### 2.3. Drug preparation

Epibrassinolide was purchased from Sigma-Aldrich Corp. (St. Louis, MO, USA). Ten mg epibrassinolide was solubilized with DMSO for a stock concentration of 5 mM.

### 2.4. Measurement of polyamine levels

The polyamine content of each sample was determined by high-pressure liquid chromatography (HPLC) (Agilent Technologies, Inc., Wilmington, DE, USA) as described by Singh et al. (1990). Briefly, 1 × 106 cells were harvested from 60 mm Petri dishes, washed with PBS, and pelleted. The cell pellet was solubilized in 50% trichloroacetic acid and centrifuged at 16,000 rcf for 20 min. The supernatant was kept and the benzoylation process was performed. Endogenous polyamine levels were determined using HPLC (Agilent Technologies, Inc., Santa Clara, CA, USA).

### 2.5. Determination of autophagy induction by acridine orange staining

Each colon cancer cell line was seeded at a density of 1 × 105 cell/well in 6-well plates. Following the treatment of cells with EBR for 48 h, the medium was carefully discarded and the samples were stained with 1 µg/mL acridine orange for 15 min. After the incubation, the cells were washed with PBS and immediately analyzed by fluorescence microscopy.

### 2.6. Determination of autophagy induction by monodansylcadaverine staining

Each colon cancer cell line was seeded at a density of 1 × 105 cell/well in 6-well plates. Following the treatment of the cells with EBR for 48 h, the medium was carefully discarded and the samples were stained with 0.05 mM monodansylcadaverine for 10 min. After the incubation, the cells were washed with PBS and immediately analyzed by fluorescence microscopy.

### 2.7. Flow cytometer analysis

Each colon cancer cell line was seeded at a density of 1 × 105 cell/well in 6-well plates. Following the treatment of the cells with EBR for 48 h, the medium was carefully discarded and the samples were stained with 1 µg/mL acridine orange for 15 min. After the incubation, the cells were collected by trypsinization and centrifugation and then were resuspended in 500 µL PBS. The cells were analyzed with a BD Accuri C6 flow cytometer. The results were shown as quadrant dot plots.

### 2.8. Statistical analysis

All the experiments presented in this study are the representative images of at least triplicate results. The statistical significance was analyzed by GraphPad Prism 6 software (GraphPad Software Inc., La Jolla, CA, USA) with Bonferroni’s multiple comparisons test ANOVA. The level of significance was taken as P < 0.05. Densitometric analyses of Western blot images were calculated by ImageJ (Rueden et al., 2017).

## 3. Results

### 3.1. Epibrassinolide induces autophagy through disruption of Akt/mTOR pathway

**Figure 1:**
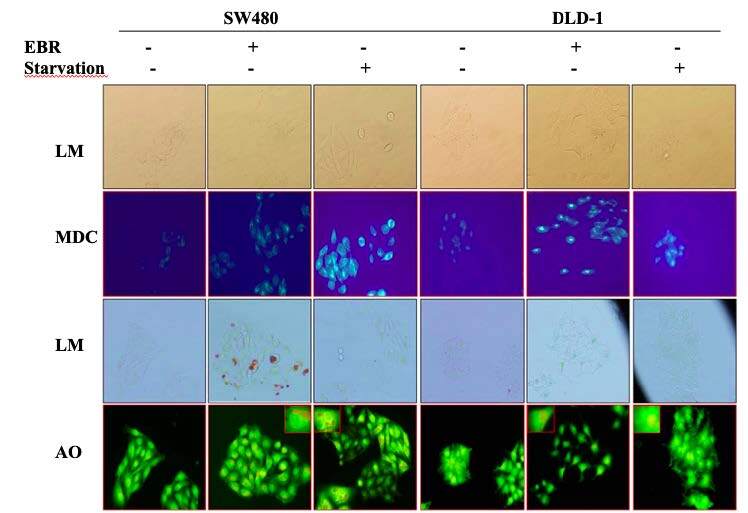
EBR altered PI3K/Akt/mTOR signaling axis in SW480 and DLD-1 colon cancer cells. A. DLD-1 and B. SW480 colon cancer cells were exposed to EBR treatment in a time-dependent manner within 48 h. Total proteins from extracted samples were analyzed for the analysis of Akt, p-Akt ser473, p-PDK ser241, mTOR, p-mTOR ser2448. b-actin was used for loading control. The band intensities of three independent immunoblotting results were calculated with ImageJ and fold ratio compared to untreated control was given below of each image with their graphs.

The ability of EBR to disrupt cancer cell homeostasis was evaluated on the human colorectal cancer cell lines SW480 and DLD-1. Disruption was shown via Western blot and the disruption was related to the Akt/mTOR pathway (Figure 1). Following the treatment of EBR, the SW480 cells doubled the expression of the total Akt protein. However, with the normalization of b-actin, no significant changes were observed for the p-Akt ser473 in a time-dependent manner. In contrast, in the DLD-1 cells, the total Akt expression diminished at the 48-h time point following the EBR treatment. In contrast, the p-Akt ser473 protein levels upregulated at any time point following the EBR treatment. The expression of the p-Akt ser473 protein was regulated by the autophosphorylation of the p-PDK1 serine241 protein (Persad et al., 2001). In the SW480 cells, there was no downregulation detected until the 48-h time point following EBR. However, in DLD-1 cells, the p-PDK1 ser241 protein expression upregulated in a time-dependent manner correlating with the p-Akt ser473 result. The mammalian target of rapamycin regulates the homeostasis of cells and consists of 2 different complexes. The mTORC1 regulates translation and disrupt by energy deprivation. The total mTOR protein was downregulated for both the SW480 and DLD-1 cells at 48 h following the EBR treatment. However, at 12 and 24 h of treatment, the total mTOR protein expression increased in the SW480 cells. The phosphorylation of mTOR at the ser2448 site regulates the activation of the mTORC1. The p-mTOR1 ser2448 protein expression almost diminished in SW480 following the EBR treatment. On the other hand, the DLD-1 cells gained the function of the p-mTORser2448 at 48 h following the EBR treatment.

### 3.2. Accumulation of autophagy-related fluorescence dyes was confirmed macroautophagy induction regulated by EBR administration

**Figure 2:**
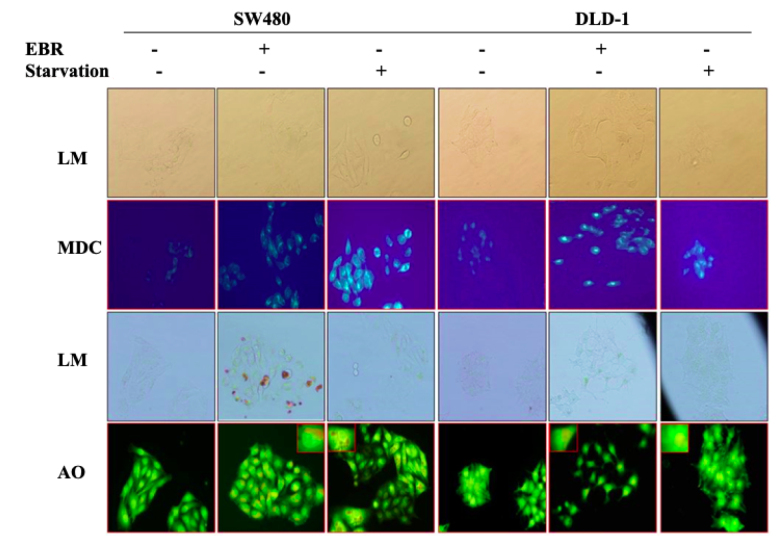
EBR is an autophagic drug in colon cancer cells. Monodansyl cadaverine (MDC), acridine orange (AO) staining for SW480 and DLD-1 cells were given following EBR treatment for 48 h EBR in treatment and results were compared to starvation. Given image was the representative one obtained from three independent experiments for each condition.

Following the loss of the mTORC1 function, autophagic alterations were revealed by MDC and AO staining (Figure 2). For staining, the 48-h time point was selected following the alterations of the Akt/mTOR pathway and starvation was used as a positive control since the energy deprivation was known to induce autophagy in various cell lines. The accumulation of MDC and AO dyes following the EBR treatment was observed by fluorescence microscopy. Both MDC and AO accumulations were increased following the EBR administration for both cell lines.

### 3.3. Spermidine administration significantly altered the macroautophagy pathway with the presence of EBR

**Figure 3:**
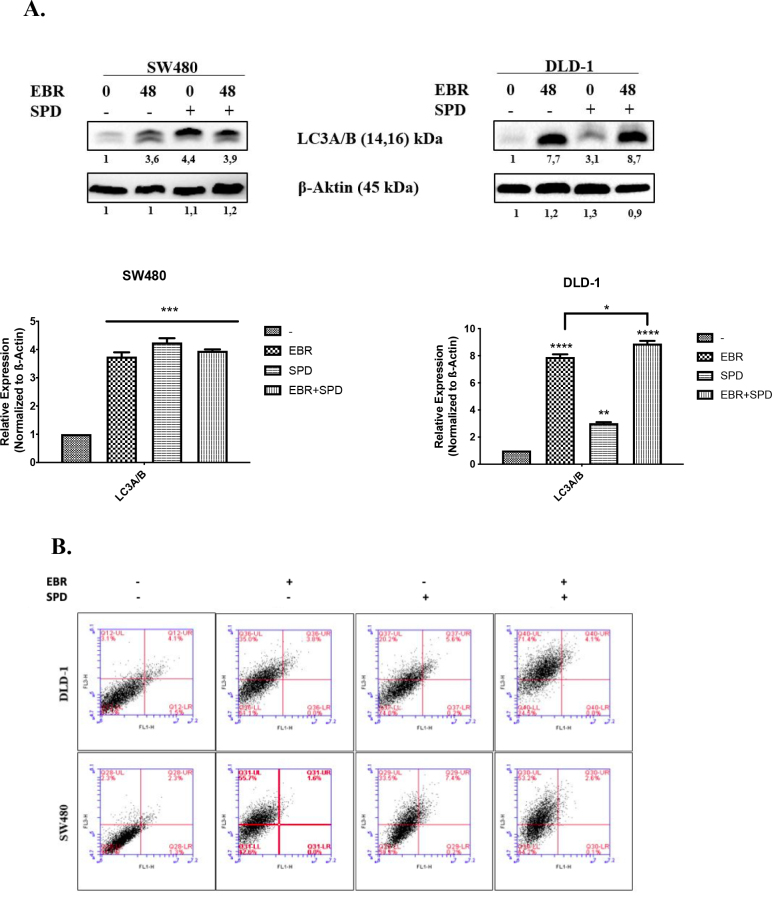
EBR triggered lipidation of LC3 in SW480 colon cancer cells. A. DLD-1 and SW480 colon cancer cells were exposed to EBR alone or SPD cotreatment in a time-dependent manner within 48 h. Total proteins from extracted samples were analyzed for the analysis of LC3. b-actin was used for loading control. The band intensities of three independent immunoblotting results were calculated with ImageJ and fold ratio compared to untreated control was given below of each image with their graphs. B. 1 × 105 cell/well in 6-well plates were used for FACS analysis of both cell lines following EBR alone and Spd cotreatment for 48 h. Autophagic vacuole formation was visualized by flow cytometry by acridine orange staining. Samples were calculated using the FL1 channel (excitation, 546 nm; emission, 575/640 nm). Following the acridine orange staining quadrants of distributed populations were analyzed. Given image is the representative one of three different experimental results.

Spd is a natural polyamine that stimulates autophagy in various cell lines. LC3 is a known autophagic marker and it is necessary for macroautophagy. We treated the cells with Spd to compare the autophagy induction effect of EBR. Then, we performed a combined treatment of Spd and EBR to check whether they act in a synergistic way in colon cancer cell lines. The combined effect of Spd and EBR on autophagy was evaluated with the detection of the LC3 protein expression and the flow cytometer analysis following AO staining. The Spd administration combined with the EBR treatment upregulated the LC3A/B protein expression compared to either the control or only the EBR treatment in the SW480 and DLD-1 cell lines (Figure 3A). However, there were no significant alterations spotted between only the EBR and Spd+EBR treatment for SW480 in the flow cytometer analysis. In contrast, the flow cytometer analysis of the DLD-1 cells was positively correlated with the alterations of the LC3A/B protein expression and significantly increased with the Spd administration and the presence of the EBR treatment (Figure 3B).

### 3.4. EBR treatment depleted intracellular polyamine levels via regulating polyamine metabolism and preventing polyamine pool retention

**Figure 4:**
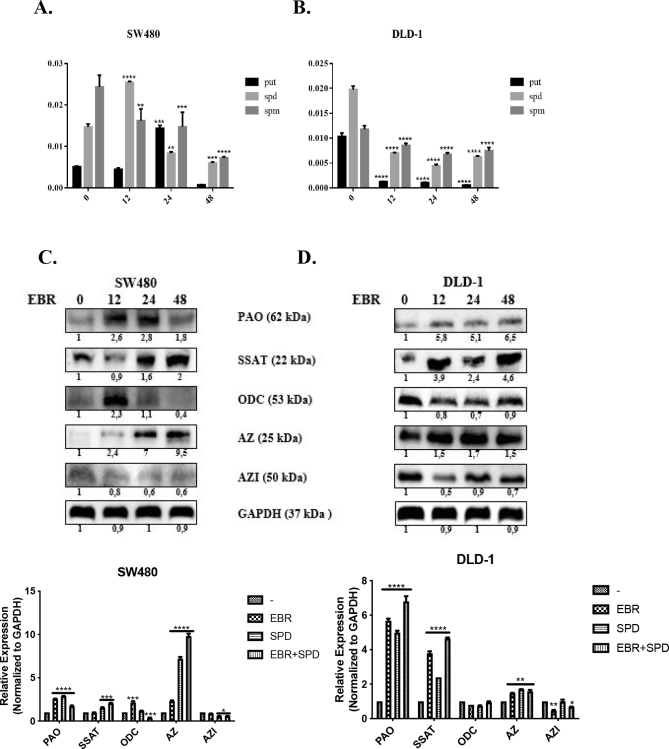
EBR altered polyamine metabolism in colon cancer cells. A. The time-dependent effect of EBR was determined for polyamine alteration following benzoylation of samples and then analysis was done by HPLC in SW480 and DLD-1 colon cancer cells. B. The protein expression profile for polyamines metabolism members SSAT, PAO, ODC, AZ, and AZI were determined by immunoblotting. Following time-dependent exposure of cells to EBR, protein extracts were run on a 12% SDS gel, which transferred onto PVDF membranes to be blotted with appropriate antibodies overnight. β-actin was used as the loading control. The given blot images were the representative one of three independent assays. ImageJ calculations were done according to the untreated sample and represented with their graphs.

EBR was further evaluated for its ability to impair cancer cell polyamine metabolism using benzoylation and HPLC analysis. The SW480 cells had converted the spermine to spermidine as a first response at 12 h of the EBR treatment. Therefore, the SW480 cells were kept converting spermidine to putrescine at the 24-h time point. However, at the 48-h time point following the EBR treatment, the polyamine pool of the SW480 cells collapsed correlating with the rest of the results. However, in the DLD-1 cells, polyamine pools were consumed in a time-dependent manner (Figure 4A). Polyamine metabolism was regulated by specific enzymes called ODC, SSAT, PAO, AZ, and AZI. These protein expressions were evaluated via Western blot (Figure 4B). The PAO protein expression was upregulated at all time points correlating with the HPLC results and the SSAT protein levels increased at 24-h and 48-h time points. The alteration of the expression of the AZ protein levels diminished the ODC protein expression at 24-h and 48-h time points, but not at 12-h time point and the AZI protein expression was downregulated in a time-dependent manner. In the DLD-1 cells, the PAO protein expression was upregulated in a time-dependent manner and theSSAT protein levels were increased at all time points compared to the control. The ODC protein expression was decreased and the AZ protein expression was upregulated at alltime points correlating with each other. Therefore, the AZI protein levels were downregulated at alltime points correlating with the AZ protein expression.

## 4. Discussion

One of the promising members of brassinosteroids is EBR, an apoptotic inducer in cancer cell lines which exerts a therapeutic potential. The majority of cancer cells show rapid responses against EBR treatment, which alter different stress-mediated cellular responses (Banerjee et al., 2019; Kuryayeva et al., 2019). In this study, we found that the EBR treatment caused the phosphorylation of the Akt ser 473 residue in the SW480 and DLD-1 colon cancer cells. It is well clarified that PI3K/Akt/mTOR is one of the leading survival-related signaling axis in the cells. The members of this signaling cascade have different interacting partners according to the cell type. Akt is the central regulator member of this pathway, which delivers signals from its upstream PI3K to downstream targets as mTOR. Phosphatidylinositol (3,4,5)-trisphosphate (PIP3) is the product of the activity of PI3K, which is activated by different membrane receptors for a variety of growth stimuli such as insulin, leptin, etc. PTEN (lipid phosphatase activity of the tumor suppressor tensin homolog on chromosome 10) as a negative regulator of the PI3K/Akt signaling reduced the growth stimuli via decreasing the PIP3 turnover from PIP2.Akt is localized at cytoplasm and can translocate plasma membrane to orchestrate cell survival-related mechanisms such as the regulation cytoskeleton network.

The phosphorylation of different residues on Akt determines its interacting partners such as FoXO family members, mTOR, or GSK3b. The full activation of Akt is required for dual activation by phosphoinositide-dependent kinase-1 (PDK1), which phosphorylates different residues of Akt isoforms. Due to the presence of upstream different stress inducers ofAkt, signal transduction at downstream cascades may cause differentiation in cellular responses. Aktacts as a fine-tuning mediator of mTOR, according to the selection of downstream targets of phosphorylated Akt (Franke, 2008). One of the mechanisms is that Akt phosphorylates TSC2 and inactivates the suppressor function of TSC1/TSC2 on the mTORC1 activity. Akt can also activate mTORC1 by directly phosphorylating PRAS40 and relieving its inhibitory effect on the complex (Palmieri et al., 2017). The activation of cellular energy sensors such as AMP-activated protein kinase (AMPK) activates the TSC1/TSC2 complex to suppress the mTORC1 activity in the cells. mTOR is a nutrient sensor mechanism in the cells, which could be triggered by amino acids, especially L-leucine-dependent glutamine transport mechanism in the cell (Jung et al., 2010). Amino acid supply in the cells may trigger mTOR on lysosomes due to the GTP-dependent Rag complex formation with mTOR (DeBerardinis et al., 2008; Menon et al., 2017). On the contrary, the mTORC2 may lead to the activation of Akt and triggers autophagy by increasing the expression levels of LC3 as a result of the Akt-FoXO3a axis under stress conditions (Liu et al., 2009). In addition, PDK2 may lead to the activation of mTORC2 to maintain Akt-mediated cellular responses (Zhang et al., 2012; Lee et al., 2013).

**Figure 5:**
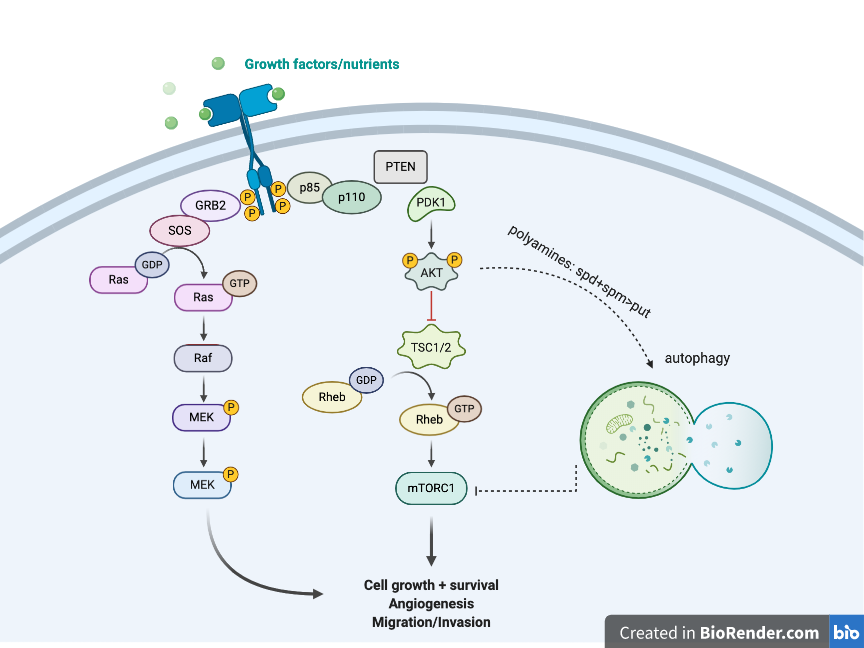
Brief presentation of EBR-mediated Akt activation in autophagy decision related to polyamines. The image was created with BioRender.

In this study, EBR increased the levels of p-Akt at ser 473 and decreased p-mTOR at ser 2448 with increased LC3 lipidation in the SW480 cells in comparison to untreated cells. In addition, positive MDC and AO staining of cells indicated that EBR induced autophagy as well as well-known autophagy inducer Spd. Although the increased levels of p-Akt at ser473 were determined in the DLD-1cells, there were fewer autophagic signatures following the EBR treatment. It is noteworthy that the depletion of the p-mTOR level was obvious within 24 h, but no longer response was observed at 48 h after the EBR treatment. In a similar way, EBR did not lead to the lipidation of LC3 48 hafter the treatment. Spermidine cotreatment enhanced the LC3 lipidation in the DLD-1 colon cancer cells as shown in previous studies (Pietrocola et al., 2015). Polyamine levels were reduced via the activation of catabolic enzymes and inactivation of polyamine uptake mechanisms in the SW480 colon carcinoma cells. These alterations led to a significant increase of the ratio of Spd+Spm/Put at 48 h following the EBR treatment in both cell lines. EBR showed similar effects in mammalian and plant cells through modulating the Spd+Spm/Put ratio under hypoxia or DNA damage-inducing stress factors. The Spd+Spm/Put ratios were increased to alleviate the stress by increasing the ATPase activity and the content of the Ca+2and Mg+2 ions (Wang et al., 2005; Obakan-Yerlikaya et al., 2017). We also confirmed the increased content of Ca+2, which increased calreticulin-mediated endoplasmic reticulum stress following the EBR treatment (Obakan et al., 2015; Obakan-Yerlikaya et al., 2017). In accordance with the previous findings, EBR reduced polyamine biosynthesis by altering ODC/antizyme interaction. We found that a significant decrease in Put levels was associated with ODC downregulation. Moreover, antizyme (AZ) upregulation was obvious due to the decreased levels of antizyme inhibitor (AZI), which might also decrease the biological function of ODC in the cells (Kahana, 2009). All these findings confirmed that EBR increased the Spd+Spm/Put levels and induced autophagic signature molecular events under the control of Akt signaling in colon cancer cells. Both cell lines showed distinct responses against EBR-mediated autophagy due to limited alteration on polyamine metabolism in the DLD-1 cells. In conclusion, further studies are required to elucidate the molecular mechanism related to different Akt-mediated responses between the SW480 and DLD-1 colon cancer cells (Figure 5). The mechanistic role of EBR may increase its therapeutic impact on colon cancer management.
